# The Bacteriophage pEp_SNUABM_08 Is a Novel Singleton Siphovirus with High Host Specificity for *Erwinia pyrifoliae*

**DOI:** 10.3390/v13071231

**Published:** 2021-06-25

**Authors:** Sang Guen Kim, Eunjung Roh, Jungkum Park, Sib Sankar Giri, Jun Kwon, Sang Wha Kim, Jeong Woo Kang, Sung Bin Lee, Won Joon Jung, Young Min Lee, Kevin Cho, Se Chang Park

**Affiliations:** 1 Laboratory of Aquatic Biomedicine, College of Veterinary Medicine and Research Institute for Veterinary Science, Seoul National University, Seoul 08826, Korea; imagine0518@snu.ac.kr (S.G.K.); giribiotek@gmail.com (S.S.G.); kjun1002@snu.ac.kr (J.K.); kasey.kim90@gmail.com (S.W.K.); kck90victory@naver.com (J.W.K.); lsbin1129@snu.ac.kr (S.B.L.); cwj0125@snu.ac.kr (W.J.J.); mushhama@snu.ac.kr (Y.M.L.); kecin6718@gmail.com (K.C.); 2 Crop Protection Division, National Institute of Agriculture Sciences, Rural Development Administration, Wanju 55365, Korea; rosalia51@korea.kr (E.R.); jungkuum@korea.kr (J.P.)

**Keywords:** singleton, bacteriophage, *Siphoviridae*, *Erwinia pyrifoliae*, *Erwinia amylovora*

## Abstract

Species belonging to the genus *Erwinia* are predominantly plant pathogens. A number of bacteriophages capable of infecting *Erwinia* have been used for the control of plant diseases such as fire blight. Public repositories provide the complete genome information for such phages, which includes genomes ranging from 30 kb to 350 kb in size. However, limited information is available regarding bacteriophages belonging to the family *Siphoviridae*. A novel lytic siphophage, pEp_SNUABM_08, which specifically infects *Erwinia pyrifoliae*, was isolated from the soil of an affected apple orchard in South Korea. A comprehensive genome analysis was performed using the *Erwinia*-infecting siphophage. The whole genome of pEp_SNUABM_08 comprised 62,784 bp (GC content, 57.24%) with 79 open reading frames. The genomic characteristics confirmed that pEp_SNUABM_08 is a singleton lytic bacteriophage belonging to the family *Siphoviridae*, and no closely related phages have been reported thus far. Our study not only characterized a unique phage, but also provides insight into the genetic diversity of *Erwinia* bacteriophages.

## 1. Introduction

*Erwinia pyrifoliae*, first isolated in the late 1990s, is regarded as the sole causative agent for blight disease in rosaceous plants in South Korea [1]. It is considered an endemic pathogen in east Asia and has been reported to cause symptoms that are indistinguishable from those observed in the fire blight disease caused by *E. amylovora* [2]. Owing to the high similarities between these species, comparative studies of the two *Erwinia* species continue to be published, even several decades after the first identification of *E. pyrifoliae* [3,4,5,6]. South Korea recently witnessed a fire blight outbreak, and the disease control protocol comprised detection, differentiation, and confirmation of pathogens, and eradication of *E.-amylovora*-infected sites [7,8]. In a few cases, both pathogenic species were detected at one site; however, the molecular techniques used for differentiation between the two pathogens are considered to be time-consuming, which poses challenges in rapid identification [7].

Bacteriophages (phages) infect their host (bacteria) via recognition of specific receptors [9]. The host specificity of phages can be utilized as a biotechnological tool, particularly for therapeutic and diagnostic purposes [10,11,12,13]. Several *Erwinia* phages have exhibited a disease (fire blight) control potency that is comparable to that of antibiotics [14,15,16]. In addition to therapeutic applications, phages have also been used for diagnosis [17,18,19]. In a representative trial, a broad-host-range *Erwinia* phage showed diagnostic potential by exhibiting higher specificity to the pathogen than that demonstrated by a commercial diagnostic strip [20]. A better understanding of phage–host interactions could help to provide valuable insights into disease control and diagnostic strategies.

The complete genomic analysis of phages is a fundamental step for understanding host–phage interactions [21,22]. A number of phages that infect *Erwinia* have been characterized and sequenced thus far. However, the majority of these phages target *E. amylovora*, and limited information is available on phages that infect *E. pyrifoliae* [23,24,25,26,27,28,29]. Moreover, the majority of *Erwinia* phages that have been studied belong to the family *Myoviridae* [30]. Thus, it is important to focus on the isolation and genomic characterization of *Erwinia* phages belonging to other families for wider biotechnological applications. In this study, we characterized a highly host-specific *Erwinia* phage (designated pEp_SNUABM_08) isolated from soil samples. 

## 2. Material and Methods

### 2.1. Phage Isolation

Phage isolation was performed as described previously, with minor modifications [23]. To isolate *E.-pyrifoliae*-infecting phages, 48 samples (31 water samples and 17 soil samples) were obtained from an area located near the region where the blight outbreak was reported in South Korea. The overnight-grown (~18 h) host culture suspension was mixed with the samples for enrichment of phages and cultured for 24 h at 27 °C. A 10-fold serial dilution of enrichment culture was spotted (10 μL) onto a lawn of bacterial culture. Samples positive for lysis zones or plaque(s) were collected to confirm their plaque-forming capacity. The samples were centrifuged, syringe-filtered (0.45 μm), and subjected to a conventional double-layer agar (DLA) assay [23]. The plaques were subcultured five times to obtain a single phage lysate.

### 2.2. Phage Propagation and Purification

Phage propagation and purification were performed using the DLA assay in accordance with a previously described protocol with minor modifications [23]. The 18 h cultured top agar was collected using SM buffer (100 mM NaCl, 50 mM Tris (pH 7.5), and 10 mM MgSO_4_) and centrifuged to isolate phages from the agar matrix, after which it was filtered through a 0.45 μm membrane filter. Precipitation using ammonium sulfate was performed for the purification of phages. The phage lysate was mixed with 2% (*v*/*v*) Tween-80, and 35% (*w*/*v*) ammonium sulfate, and was incubated for 15 min at 4 °C. The pellicle was resuspended in SM buffer and purified using CsCl gradient ultracentrifugation [23]. The high titer of phage suspension (>10^10^ plaque-forming units (PFUs)/mL) dialyzed using 7000 MWCO membrane was stored at 4 °C.

### 2.3. Transmission Electron Microscopy

The phage suspension (10 μL) was spotted on a copper grid and incubated for 1 min. Excess solution was removed, followed by the conduction of staining with uranyl acetate (2%) for 30 s. The dried grid was examined using the Talos L 120C (FEI, OR, USA) transmission electron microscope (120 kV). Dimensions of the phage virions were analyzed by measuring three independent particles.

### 2.4. Host Range Analysis

A total of 55 strains including *Erwinia* spp. (26 *E. amylovora* strains and 25 *E. pyrifoliae* strains) isolated from the blight-affected plant tissue were examined for host range analysis using the spot method [23]. The presence of inhibition zone or plaque(s) on the spotted area was considered to denote susceptibility; infectivity was represented as + and − for susceptible and nonsusceptible cases, respectively. The strains that showed an inhibition zone were further assayed using the double-layer method to exclude abortive infection, using 10-fold serial dilutions of the phage suspension. Genotyping of the *Erwinia* strains used in the present study was performed using ERIC primers: ERIC_F-3′-ATGTAAGCTCCTGGGGATTCAC-5′, ERIC_R-3′-AAGTAAGTGACTGGGGTGAGCG-5′. PCR reactions were carried out with 500 ng of genomic DNA of the strains and consisted of a denaturation at 95 °C for 3 min, followed by 35 cycles at 94 °C for 1 min, 50 °C for 1 min, and 72 °C for 5 min, and a final extension at 72 °C for 10 min.

### 2.5. Adsorption and One-Step Growth Curve

The adsorption [31] and one-step growth [32] assays were conducted as described previously. The host strain (exponential phase; ~10^8^ CFU/mL) was mixed with the phage at a multiplicity of infection (MOI) of 0.001 and cultured at 27 °C. For the adsorption assay, samples were collected at 1, 3, 5, 10, 20, 30, 40, 50, and 60 min after incubation. The PFU was measured using the supernatant obtained after centrifugation for the enumeration of unadsorbed phages. For the one-step growth assay, after phage adsorption for 50 min (>95% adsorption rate), unadsorbed phages were discarded after centrifugation. Then, the pellet was resuspended with prewarmed broth and samples were acquired every 20 min for a timeframe of 180 min to analyze the latent period and burst size.

### 2.6. DNA Extraction and Sequencing

Phage DNA isolation was performed using the phenol–chloroform method [23]. Briefly, 1 mL of the phage suspension (>10^10^ PFU/mL) was mixed with 10 U of DNase I and RNase A and incubated for 1 h at 37 °C. After the addition of 0.5 M of ethylenediaminetetraacetic acid for the inhibition of enzymes, the suspension was subjected to treatment with proteinase K for 3 h at 56 °C. Next, DNA was isolated using a phenol:chloroform:isoamylalchol (25:24:1) solution and precipitated with ethanol. Sequencing and assembly of the isolated DNA were performed using the Illumina HiSeq system (Illumina, San Diego, CA, USA) at Macrogen (Seoul, South Korea).

### 2.7. Genome Analysis

Open reading frame (ORF) identification was performed using GeneMarkS and the Rapid Annotation using Subsystem Technology (RAST) server [33,34]. The putative function of each ORF was predicted and annotated using protein BLAST. The tRNA, antibiotic resistance-related genes, and virulence-related genes were identified using tRNAscan-SE (v2.0), ResFinder (v3.2), and the VirulenceFinder (v2.0) server, respectively [35,36,37]. A total of 72 complete genome sequences of *Erwinia* phage spp. available in the NCBI nucleotide database were included for comparative genome analysis. Comparative genome analysis was performed using Coregene3.5 (blastp threshold: 75), Gepard (word size: 10), and Easyfig (E-value cut off: 0.001) software [38,39,40]. The phylogenetic tree was generated using Mega X with maximum likelihood method (1000 bootstrap) Virus Classification and Tree Building Online Resource (VICTOR) with the recommended settings for prokaryotic viruses [41,42].

### 2.8. Proteome Analysis

Mass spectrometric analysis of the structural proteins of phage pEp_SNUABM_08 was performed as described [23]. The phage proteins prepared by implementing five rounds of freeze–thaw cycles were separated using 12% SDS-PAGE. Subsequently, the proteins spanning the entire lane were in-gel digested with trypsin (100 ng/μL). The resultant proteins derived from pEp_SNUABM_08 were identified by using a nano high-resolution LC-MS/MS spectrometer Q Exactive Hybrid Quadrupole-Orbitrap (Thermo Scientific, MA, USA) equipped with the Dionex U 3000 RSLC nano HPLC system.

## 3. Results

### 3.1. Biological Analysis of pEp_SNUABM_08

**Figure 1:**
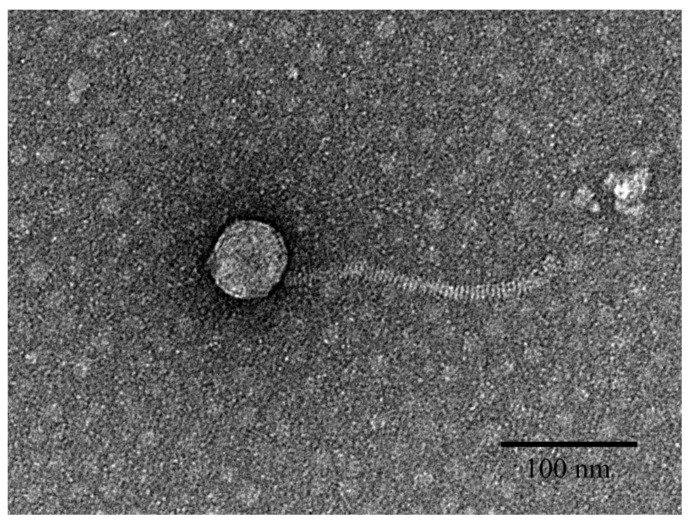
Transmission electron microscopy result showing the structure of *Erwinia pyrifoliae* phage pEp_SNUABM_08. Scale bar: 100 nm.

The *Erwinia* phage pEp_SNUABM_08 was isolated from the soil of a blight-affected apple orchard (located on Jecheon, Chungcheongbuk province) using *E. pyrifoliae* (KACC13945) as an indicator strain. Morphological analysis revealed that the phage belonged to the Siphovirus family. The phage pEp_SNUABM_08 possessed an icosahedral head (62 ± 4 nm in diameter) and a long noncontractile tail (190 ± 12 nm in length) (Figure 1). 

The host range assay was performed against 51 *Erwinia* strains, including *E. amylovora* and *E. pyrifoliae*, isolated from blight-affected plant tissues around South Korea in 1999, 2019, and 2020, and four other strains belonging to three bacterial species (Table 1). The phage pEp_SNUABM_08 showed highly specific infectivity. Of 26 *E. amylovora* strains, phage pEp_SNUABM_08 could infect only two strains, YKB 14748 and RA0030, isolated from Chungcheongbuk and Gyeonggi provinces. Of 25 *E. pyrifoliae* strains, the phage pEp_SNUABM_08 mainly infected strains isolated from Gangwon province (11/15 strains) having different clonal lineages, and 1 strain isolated from Gyeongsangbuk province, which showed a geographical spread (Figure S1). Lysis from without was not observed and plaques were obtained for all susceptible strains in case of low-concentration phage suspension(s).

**Figure 2:**
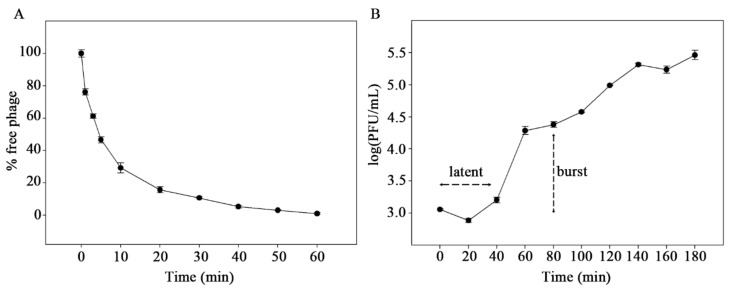
Adsorption (**A**) and one-step growth curve (**B**) of pEp_SNUAB_08.

The phage pEp_SNUABM_08 showed a low adsorption rate, which reached 70%, 90%, and 95% at 10, 30, and 40 min, respectively (Figure 2A). The first burst of phages commenced at 40 min (latent period) after inoculation with the burst size of 20 PFU/cell and the second burst was observed at 100 min after inoculation (Figure 2B).

### 3.2. Protein Analysis of pEp_SNUABM_08

Upon analyzing the profiles of structural proteins of pEp_SNUABM_08, we identified 12 structural proteins including 10 generally identified structural proteins including head-to-tail joining protein (gp12), portal protein (gp13), major capsid protein (gp16), tape measure protein (gp24), and two distinct proteins (Ig-like domain-containing protein; gp21, and head decoration protein; gp15), which were also predicted in the genome analysis (Table 2).

### 3.3. Genomic Analysis of pEp_SNUABM_08

**Figure 3:**
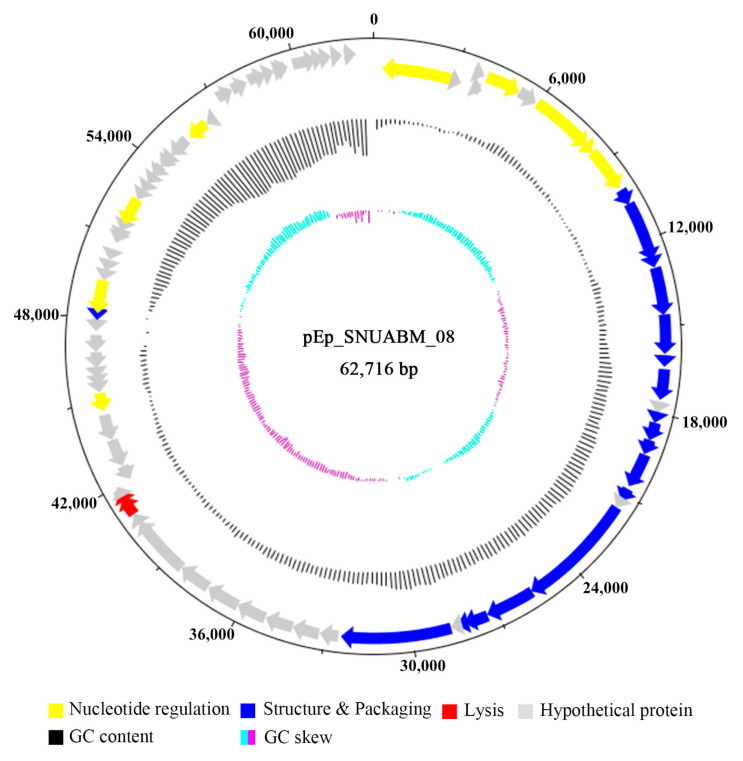
Complete genome map of pEp_SNUABM_08. The ORFs were categorized and color-coded based on their function; structure and packaging, blue; nucleotide regulation, yellow; lysis, red; hypothetical protein, gray. Scale = base pair.

pEp_SNUABM_08 harbors a 62,715 bp dsDNA genome (GC content, 57.24%). Seventy-nine ORFs were detected in the genome; however, tRNA genes were not detected. Among the predicted ORFs, 48 were located on the positive strand and 31 were located on the negative strand (Figure 3). The predicted ORFs were categorized into the following four groups based on their function: nucleotide regulation (e.g., Cas4-like protein, putative exonuclease), structure and packaging (e.g., putative tail fiber protein, terminase small/large subunit), lysis (e.g., lysis protein A like protein, Rz like protein), and hypothetical proteins. The majority of the ORFs were found to be for hypothetical proteins (57 ORFs; 72.15%); lysogeny-, antibiotic-resistance-, and bacterial-virulence-related genes were not detected (Table S1). 

**Figure 4:**
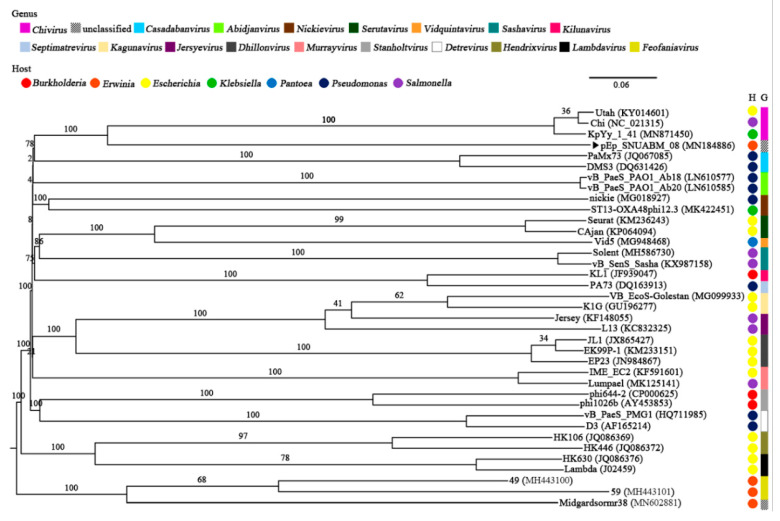
Whole-genome phylogenetic analysis of *Siphoviridae* phages infecting members of the class Gammaproteobacteria. The pEp_SNUABM_08 phage isolated in the present study is highlighted using a black arrow (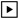). The genera (*Chivirus*, magenta; unclassified, mosaic; *Casadabanvirus*, cyan; *Abidjanvirus*, light green; *Nickievirus*, brown; *Serutavirus*, dark green; *Vidquintavirus*, orange; *Sashavirus*, turquoise; *Kilunavirus*, crimson; *Septimavirus*, sky blue; *Kagunavirus*, bisque; *Jersyevirus*, violet; *Dhillonvirus*, kakhi; *Murrayvirus*, petal; *Stanboltvirus*, gray; *Detrevirus*, white; *Hendrixvirus*, chartreuse; *Lambdavirus*, black; *Feofaniavirus*, chrome yellow) and host (*Burkholderia*, red; *Erwinia*, orange; *Escherichia*, yellow; *Klebsiella*, green; *Pantoea*, blue; *Pseudomonas*, dark blue; *Salmonella*, purple) are indicated using respective colors.

**Figure 5:**
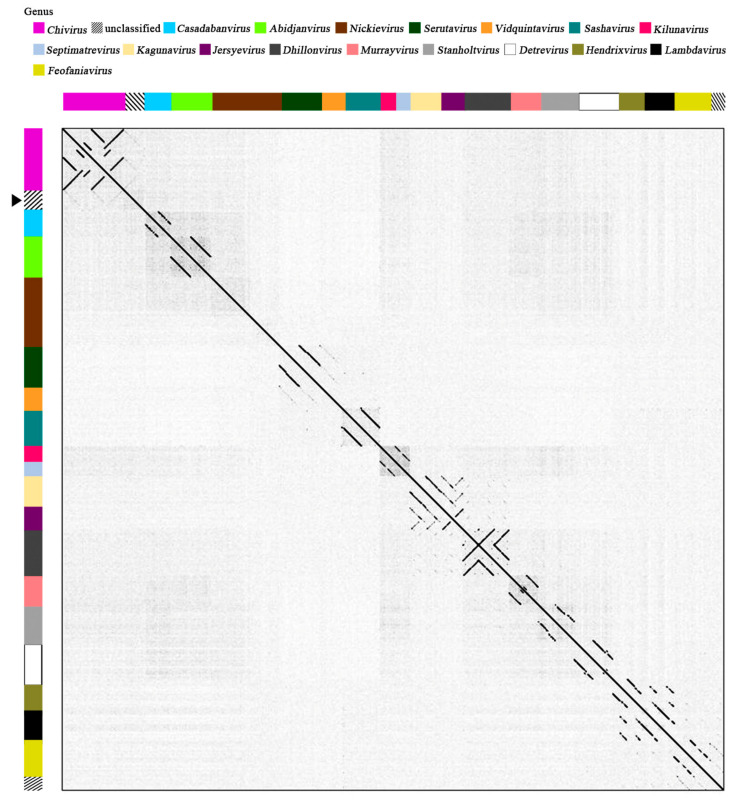
Comparative analysis of whole genome sequences of the phage pEp_SNUABM_08 and related phages using dot plot. The pEp_SNUABM_08 phage isolated in the present study is highlighted using a black arrow (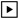). The arrangement of sequences was paralleled with the whole-genome sequence phylogeny (Figure 4).

The predicted genes showed best matches with those of phages classified under the genus *Chivirus* with a relatively low identity (23–76%). The genome organization of pEp_SNUABM_08 was slightly similar to that of the well-known lytic bacteriophage Chi; however, a strong homology could not be observed for the last section of the genome (~37 kb; Figure S2). As expected, the complete nucleotide sequence of pEp_SNUABM_08 did not form a cluster with phages of the family *Siphoviridae,* which infect bacterial strains of class Gammaproteobacteria (*Burkholderia, Erwinia, Escherichia, Klebsiella, Pantoea, Pseudomonas,* and *Salmonella*), as shown in the phylogenetic tree (Figure 4), and did not exhibit the generation of a strong line in the dot plot analysis (Figure 5). 

**Figure 6:**
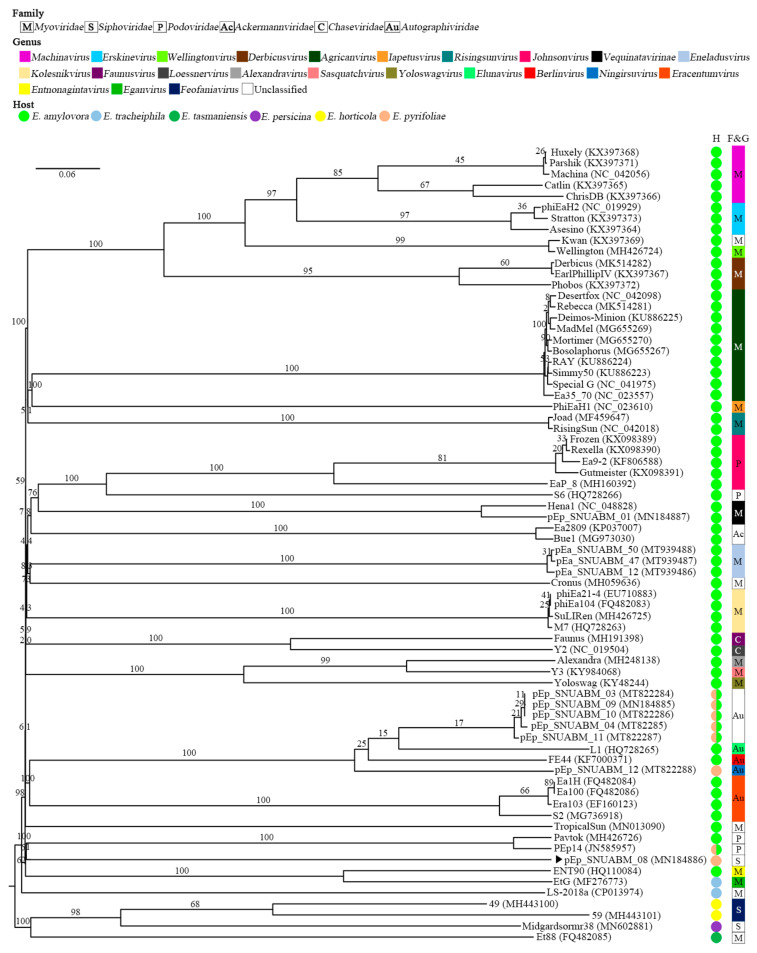
Phylogenetic analysis of *Erwinia* phages conducted using the VICTOR software. The pEp_SNUABM_08 phage isolated in the present study is highlighted using a black arrow (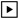). The numbers above the branches represent Genome-BLAST Distance Phylogeny (GBDP) pseudo-bootstrap support values based on the conduction of 100 replications.

A core gene analysis was performed to conduct a more detailed comparation. The replication strategy of *Erwinia* phage pEp_SNUABM_08 was similar to that of Chi, as the major component phage proteins (41/79; 51.9%) encoding structural, packaging, nucleotide regulation, or lysis proteins were conserved. In contrast, the hypothetical proteins encoded by the last section of their genomes were unique to each phage. The proteins are considered to endow pEp_SNUABM_08 with the singleton status. Moreover, phylogenetic analysis among *Erwinia* phages showed the existence of a sole cluster of pEp_SNUABM_08 (Figure 6). Only one core protein was shared among *Siphoviridae* phages infecting *Erwinia* spp. (pEp_SNUABM_08, 49, 59, Midgardsormr38), namely the tape measure protein.

**Figure 7:**
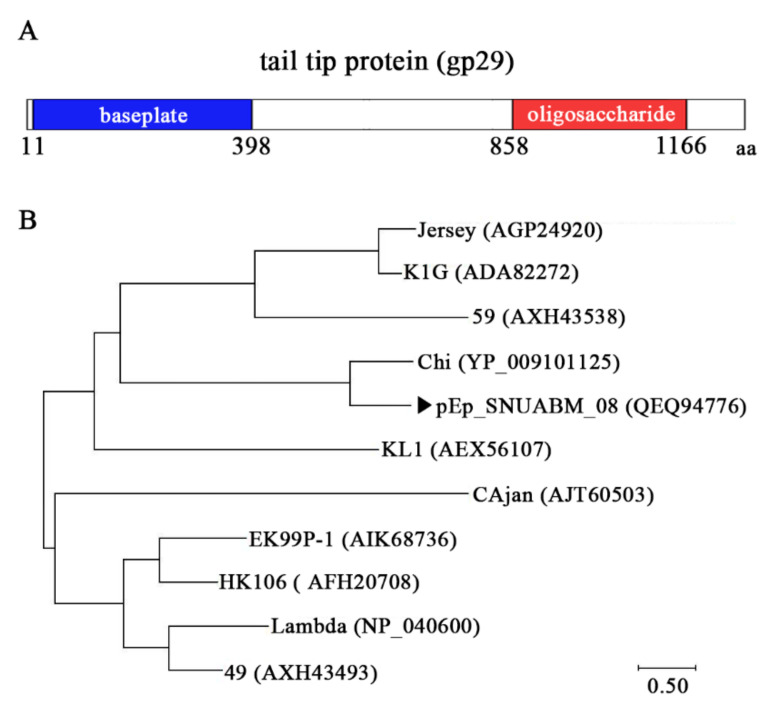
Schematic representation of the tail tip protein (gp29) of pEp_SNUABM_08 (**A**). The N-terminal (baseplate binding domain), and C-terminal (oligosaccharide binding domain) are colored in blue and red, respectively. Phylogeny analysis of the tail tip protein of pEp_SNUABM_08 (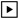) and related phages (**B**).

The structure of the tail tip protein (gp29) of pEp_SNUABM_08 comprised an N-terminal baseplate binding domain and a C-terminal oligosaccharide binding domain (Figure 7A). The N-terminal domain showed homology with phage-originated domains such as T5 baseplate hub (Q6QGE9), Lambda Tip attachment protein J (P03749), and phage-tail 3 (PF13550.7). However, the C-terminal of gp29 matched with the binding domain of the gene transfer agent of *Rhodobacter capsulatus* (6TEH). Additionally, gp29 of pEp_SNUABM_08 did not show clustering with other closely related phages, as shown in Figure 7B.

## 4. Discussion

The first step in this study was the isolation of phages with different host ranges and analysis of their genome sequences. In this study, we isolated the *Erwinia* phage pEp_SNUABM_08. Although most reported *Erwinia* phages exhibit morphological characteristics similar to those of myoviruses or podoviruses, pEp_SNUABM_08 is a distinct siphovirus-shaped virion. It exhibited high host specificity against the *E. pyrifoliae* strains (Table 1) compared to the myophages or podophages that were isolated from South Korea. Nevertheless, pEp_SNUABM_08 may possess biocontrol and diagnostic potential for black shoot disease, which has been previously reported in Gangwon province, South Korea [23,43,44]. 

The host specificity of pEp_SNUABM_08 may be associated with its atypical tail protein, which is responsible for host recognition [45,46]. In particular, it has been elucidated that the host receptor-binding protein (RBP) is located at the tip of the tail fiber protein, which is located in the C-terminal end of the protein [47,48]. The arrangement of tail fiber genes in the lytic phage pEp_SNUABM_08 is similar to that observed in phage lambda, as they harbor the lambda-like tail fiber domains, namely tail completion protein Z (gp19), tail tube terminator protein U (gp20), tail tip protein M (gp25), tail tip protein L (gp26), tail tip assembly protein I (gp27), and tail tip attachment protein J (gp29) [49]. The tail tip attachment protein J is considered to play a major role in host recognition, and the tail tip protein of pEp_SNUABM_08 (gp29) also comprises the N-terminal baseplate binding domain, C-terminal RBP, and oligosaccharide binding domain (Figure 7A). However, the C-terminal domain of pEp_SNUABM_08 did not exhibit strong homology with any of the phage-originated RBPs, and gp29 was solely clustered among closely related phage tail proteins (Figure 7B). This single clustering characteristic may explain the host specificity of pEp_SNUABM_08. 

Many years have passed after its initial isolation, and phages that are closely related to phage pEp_SNUABM_08 have not been reported. In addition, the majority (72%) of its genome encoded hypothetical proteins; many proteins did not exhibit homology with other proteins available in the database. The distinct biological and genomic characteristics of *Erwinia* pEp_SNUABM_08 are considered to originate from its singleton status. Further studies focusing on structural and functional characterization of this virus may provide insights into novel phage–host interaction mechanisms, especially for *Erwinia* phages.

## Figures and Tables

**Table 1:** Host range of *Erwinia* phage pEp_SNUABM_08.

Species	Strain	Isolated		Infectivity ^a^
		Year	Province	
*Erwinia amylovora*	YKB 14715	2019	Chungcheongbuk	−
	YKB 14740	2019	Chungcheongbuk	−
	YKB 14742	2019	Chungcheongbuk	−
	YKB 14748	2019	Chungcheongbuk	+
	YKB 14750	2019	Chungcheongbuk	−
	RA0030	2020	Gyeonggi	+
	RA0031	2020	Gyeonggi	−
	RA0032	2020	Gyeonggi	−
	RA0033	2020	Gyeonggi	−
	RA0034	2020	Gyeonggi	−
	RA0035	2020	Gyeonggi	−
	RA0041	2019	Chungcheongnam	−
	RA0042	2020	Chungcheongnam	−
	RA0043	2020	Chungcheongnam	−
	RA0044	2020	Chungcheongnam	−
	RA0045	2020	Chungcheongbuk	−
	RA0051	2020	Chungcheongbuk	−
	RA0052	2020	Chungcheongbuk	−
	RA0053	2020	Chungcheongbuk	−
	RA0054	2020	Chungcheongbuk	−
	RA0055	2020	Chungcheongbuk	−
	RA0062	2020	Chungcheongbuk	−
	RA0063	2020	Chungcheongbuk	−
	RA0064	2020	Chungcheongbuk	−
	RA0065	2020	Chungcheongbuk	−
	RA0066	2020	Chungcheongbuk	−
*Erwinia pyrifoliae*	KACC13945	1999	Gangwon	+
	KACC13946	1999	Gangwon	+
	KACC13948	1999	Gangwon	+
	KACC13949	1999	Gangwon	+
	KACC13952	1999	Gangwon	+
	RP0100	2020	Gangwon	+
	RP0101	2020	Gangwon	+
	RP0102	2020	Gangwon	+
	RP0103	2020	Gangwon	−
	RP0104	2020	Gangwon	+
	RP0105	2020	Gangwon	+
	RP0108	2020	Gangwon	−
	RP0109	2020	Gangwon	−
	RP0110	2020	Gangwon	−
	RP0111	2020	Gyeonggi	−
	RP0112	2020	Gyeonggi	−
	RP0113	2020	Gyeonggi	−
	RP0114	2020	Gyeongsangbuk	−
	RP0115	2020	Gyeongsangbuk	+
	RP0116	2020	Chungcheongbuk	−
	RP0117	2020	Chungcheongbuk	−
	RP0118	2020	Chungcheongbuk	−
	RP0119	2020	Chungcheongbuk	−
	RP0120	2020	Gangwon	+
	RP0121	2020	Chungcheongbuk	−
*Pectobacterium carotovorum*	KACC17004	N/A	Gangwon	−
	KACC18645	N/A	Gangwon	−
*Pseudomonas aeruginosa*	KCCM40395	N/A	N/A	−
*Escherichia coli*	KCTC2571	N/A	N/A	−

^a^ + and − indicate susceptible and nonsusceptible strains, respectively.

**Table 2:** Profiles of LC-MS/MS-detected structural proteins of *Erwinia* phage pEp_SNUABM_08.

Protein	Putative Function	Molecular Weight (kDa)	Number of Identified Peptides	Coverage (%)
gp12	Head-to-tail joining protein	9.6	11	28
gp13	Portal protein	61.2	100	25
gp15	Head decoration protein	14.2	2	19
gp16	Major capsid protein	39.9	90	36
gp21	Ig-like domain-containing protein	41.9	58	11
gp22	Putative tail assembly chaperone	17.3	127	40
gp24	Tape measure protein	148.4	528	48
gp25	Distal tail protein	64.7	258	21
gp29	Putative tail protein	139.1	218	25
gp30	Putative tail fiber protein	23.7	5	10
gp38	Virion protein	25.2	106	38
gp51	Tail fiber protein	9.2	10	47

## Data Availability

The whole genome sequence of the phage pEp_SNUABM_08 has been deposited at GenBank under the accession number MN184886.

## Supplementary Materials

The following are available online at https://www.mdpi.com/article/10.3390/v13071231/s1, Table S1: The profile of ORFs in *Erwinia* phage pEp_SNUABM_08; Figure S1: ERIC-PCR genotyping of *Erwinia* spp. used in this study for host range analysis; Figure S2: Comparative whole genome analysis of *Erwinia* phage pEp_SNUABM_08 and *Salmonella* phage Chi.

## References

[1] Kim W.S., Gardan L., Rhim S.L., Geider K. (1999). *Erwinia pyrifoliae* sp. nov., a novel pathogen that affects Asian pear trees (*Pyrus pyrifolia Nakai*). Int. J. Syst. Evol. Microbiol..

[2] Shrestha R., Koo J.H., Park D.H., Hwang I., Hur J.H., Lim C.K. (2003). *Erwinia pyrifoliae*, a causal endemic pathogen of shoot blight of Asian pear. Plant Pathol. J..

[3] Kim W.S., Jock S., Paulin J.P., Rhim S.L., Geider K. (2001). Molecular detection and differentiation of *Erwinia pyrifoliae* and host range analysis of the Asian pear pathogen. Plant Dis..

[4] Zhao Y., Qi M. (2011). Comparative genomics of *Erwinia amylovora* and related Erwinia species—What do we learn?. Genes.

[5] Gehring I., Geider K. (2012). Differentiation of *Erwinia amylovora* and *Erwinia pyrifoliae* strains with single nucleotide polymorphisms and by synthesis of dihydrophenylalanine. Curr. Microbiol..

[6] Lee G.M., Ko S., Kim D., Oh C.S. Comparative genomic analysis reveals an evolutionary trace in the genome of *Erwinia pyrifoliae*, a black shoot blight pathogen. Proceedings of the 2019 KSBB Fall Meeting and International Symposium.

[7] Park D.H., Lee Y.G., Kim J.S., Cha J.S., Oh C.S. (2017). Current status of fire blight caused by *Erwinia amylovora* and action for its management in Korea. J. Plant Pathol..

[8] Lee M.H., Ji S., Ham H.H., Kong G.H., Park D.S., Lee Y.H. (2020). First report of fire blight of Apricot (*Prunus armeniaca*) caused by *Erwinia amylovora* in Korea. Plant Dis..

[9] Bertozzi Silva J., Storms Z., Sauvageau D. (2016). Host receptors for bacteriophage adsorption. FEMS Microbiol. Lett..

[10] Santos S.B., Azeredo J. (2019). Bacteriophage-based biotechnological applications. Viruses.

[11] Melo L.D., Oliveira H., Pires D.P., Dabrowska K., Azeredo J. (2020). Phage therapy efficacy: A review of the last 10 years of preclinical studies. Crit. Rev. Microbiol..

[12] Kakasis A., Panitsa G. (2019). Bacteriophage therapy as an alternative treatment for human infections. A comprehensive review. Int. J. Antimicrob. Agents.

[13] Meile S., Kilcher S., Loessner M.J., Dunne M. (2020). Reporter phage-based detection of bacterial pathogens: Design guidelines and recent developments. Viruses.

[14] Nagy J., Király L., Schwarczinger I. (2012). Phage therapy for plant disease control with a focus on fire blight. Open Life Sci..

[15] Boulé J., Sholberg P.L., Lehman S.M., O’gorman D.T., Svircev A.M. (2011). Isolation and characterization of eight bacteriophages infecting *Erwinia amylovora* and their potential as biological control agents in British Columbia, Canada. Can. J. Plant Pathol..

[16] Schwarczinger I., Nagy J.K., Künstler A., Szabó L., Geider K., Király L., Pogány M. (2017). Characterization of *Myoviridae* and *Podoviridae* family bacteriophages of *Erwinia amylovora* from Hungary-potential of application in biological control of fire blight. Eur. J. Plant Pathol..

[17] Schofield D.A., Bull C.T., Rubio I., Wechter W.P., Westwater C., Molineux I.J. (2012). Development of an engineered bioluminescent reporter phage for detection of bacterial blight of crucifers. Appl. Environ. Microbiol..

[18] Van der Merwe R.G., Van Helden P.D., Warren R.M., Sampson S.L., Van Pittius N.G. (2014). Phage-based detection of bacterial pathogens. Analyst.

[19] Vu N.T., Oh C.S. (2020). Bacteriophage usage for bacterial disease management and diagnosis in plants. Plant Pathol. J..

[20] Born Y., Fieseler L., Thöny V., Leimer N., Duffy B., Loessner M.J. (2017). Engineering of bacteriophages Y2::*dpoL1-C* and Y2::*luxAB* for efficient control and rapid detection of the fire blight pathogen, *Erwinia amylovora*. Appl. Environ. Microbiol..

[21] Parmar K.M., Gaikwad S.L., Dhakephalkar P.K., Kothari R., Singh R.P. (2017). Intriguing interaction of bacteriophage-host association: An understanding in the era of omics. Front. Microbiol..

[22] Li M., Wang Y., Li F., Zhao Y., Liu M., Zhang S., Bin Y., Smith A.I., Webb G., Li J. (2020). A deep learning-based method for identification of bacteriophage-host interaction. IEEE/ACM Trans. Comput. Biol. Bioinform..

[23] Kim S.G., Lee S.B., Giri S.S., Kim H.J., Kim S.W., Kwon J., Park J., Roh E., Park S.C. (2020). Characterization of novel *Erwinia amylovora* jumbo bacteriophages from *Eneladusvirus* genus. Viruses.

[24] Besarab N.V., Akhremchuk A.E., Zlatohurska M.A., Romaniuk L.V., Valentovich L.N., Tovkach F.I., Lagonenko A.L., Evtushenkov A.N. (2020). Isolation and characterization of Hena1–a novel *Erwinia amylovora* bacteriophage. FEMS Microbiol. Lett..

[25] Sharma R., Pielstick B.A., Bell K.A., Nieman T.B., Stubbs O.A., Yeates E.L., Baltrus D.A., Grose J.H. (2019). A novel, highly related jumbo family of bacteriophages that were isolated against *Erwinia*. Front. Microbiol..

[26] Sharma R., Berg J.A., Beatty N.J., Choi M.C., Cowger A.E., Cozzens B.J.R., Duncan S.G., Fajardo C.P., Ferguson H.P., Galbraith T. (2019). Genome sequences of nine *Erwinia amylovora* bacteriophages. Microbiol. Resour. Announc..

[27] Arens D.K., Brady T.S., Carter J.L., Pape J.A., Robinson D.M., Russell K.A., Staley L.A., Stettler J.M., Tateoka O.B., Townsend M.H. (2018). Characterization of two related *Erwinia myoviruses* that are distant relatives of the PhiKZ-like jumbo phages. PLoS ONE.

[28] Müller I., Lurz R., Kube M., Quedenau C., Jelkmann W., Geider K. (2011). Molecular and physiological properties of bacteriophages from North America and Germany affecting the fire blight pathogen *Erwinia amylovora*. Microb. Biotechnol..

[29] Lehman S.M., Kropinski A.M., Castle A.J., Svircev A.M. (2009). Complete genome of the broad-host-range *Erwinia amylovora* phage ΦEa21-4 and its relationship to *Salmonella* phage Felix O1. Appl. Environ. Microbiol..

[30] Thompson D.W., Casjens S.R., Sharma R., Grose J.H. (2019). Genomic comparison of 60 completely sequenced bacteriophages that infect *Erwinia* and/or *Pantoea* bacteria. Virology.

[31] Lu Z., Breidt F., Fleming H.P., Altermann E., Klaenhammer T.R. (2003). Isolation and characterization of a *Lactobacillus plantarum* bacteriophage, ΦJL-1, from a cucumber fermentation. Int. J. Food Microbiol..

[32] Kim S.G., Jun J.W., Giri S.S., Yun S., Kim H.J., Kim S.W., Kang J.W., Han S.J., Jeong D., Park S.C. (2019). Isolation and characterisation of pVa-21, a giant bacteriophage with anti-biofilm potential against *Vibrio alginolyticus*. Sci. Rep..

[33] Besemer J., Lomsadze A., Borodovsky M. (2001). GeneMarkS: A self-training method for prediction of gene starts in microbial genomes. Implications for finding sequence motifs in regulatory regions. Nucleic Acids Res..

[34] Aziz R.K., Bartels D., Best A.A., De Jong M., Dis T., Edward R.A., Formsma K., Gerdes S., Glass E.M., Kubal M. (2008). The RAST Server: Rapid annotations using subsystems technology. BMC Genom..

[35] Lowe T.M., Eddy S.R. (1997). tRNAscan-SE: A program for improved detection of transfer RNA genes in genomic sequence. Nucleic Acids Res..

[36] Zankari E., Hasman H., Cosentino S., Vestergaard M., Rasmussen S., Lund O., Aarestrup F.M., Larsen M.V. (2012). Identification of acquired antimicrobial resistance genes. J. Antimicrob. Chemother..

[37] Joensen K.G., Scheutz F., Lund O., Hasman H., Kaas R.S., Nielsen E.M., Aarestrup F.M. (2014). Real-time whole-genome sequencing for routine typing, surveillance, and outbreak detection of verotoxigenic *Escherichia coli*. J. Clin. Microbiol..

[38] Turner D., Reynolds D., Seto D., Mahadevan P. (2013). CoreGenes3.5: A webserver for the determination of core genes from sets of viral and small bacterial genomes. BMC Res. Notes.

[39] Krumsiek J., Arnold R., Rattei T. (2007). Gepard: A rapid and sensitive tool for creating dotplots on genome scale. Bioinformatics.

[40] Sullivan M.J., Petty N.K., Beatson S.A. (2011). Easyfig: A genome comparison visualizer. Bioinformatics.

[41] Kumar S., Stecher G., Li M., Knyaz C., Tamura K. (2018). MEGA X: Molecular evolutionary genetics analysis across computing platforms. Mol. Biol. Evol..

[42] Meier-Kolthoff J.P., Göker M. (2017). VICTOR: Genome-based phylogeny and classification of prokaryotic viruses. Bioinformatics.

[43] Lim S.R., Kim I.K. (2013). New Bacteriophage φEp14.

[44] Park J., Lee G.M., Kim D., Park D.H., Oh C.S. (2018). Characterization of the lytic bacteriophage phiEaP-8 effective against both *Erwinia amylovora* and *Erwinia pyrifoliae* causing severe diseases in apple and pear. Plant Pathol. J..

[45] Davidson A.R., Cardarelli L., Pell L.G., Radford D.R., Maxwell K.L., Rossmann M.G., Rao V.B. (2012). Long noncontractile tail machines of bacteriophages. Viral Molecular Machines.

[46] Islam M.Z., Fokine A., Mahalingam M., Zhang Z., Garcia-Doval C., Van Raaij M.J., Rossmann M.G., Rao V.B. (2019). Molecular anatomy of the receptor binding module of a bacteriophage long tail fiber. PLoS Pathog..

[47] Goulet A., Spinelli S., Mahony J., Cambillau C. (2020). Conserved and diverse traits of adhesion devices from *Siphoviridae* recognizing proteinaceous or saccharidic receptors. Viruses.

[48] Pell L.G., Gasmi-Seabrook G.M., Morais M., Neudecker P., Kanelis V., Bona D., Donaldson L.W., Edwards A.M., Howell P.L., Davidson A.R. (2010). The solution structure of the C-terminal Ig-like domain of the bacteriophage λ tail tube protein. J. Mol. Biol..

[49] Rajagopala S.V., Casjens S., Uetz P. (2011). The protein interaction map of bacteriophage lambda. BMC Microbiol..

