# Supplementary material for: The Bacteriophage pEp_SNUABM_08 Is a Novel Singleton Siphovirus with High Host Specificity for Erwinia pyrifoliae
Source: Viruses. 2021 Jun 25;13(7):1231. doi: 10.3390/v13071231 (PMC8310351; doi:10.3390/v13071231)
Supplement: Supplementary file 1 [file viruses-13-01231-s001.zip › viruses-1223700-supplementary.pdf]

**Supplementary information**

**The bacteriophage pEp_SNUABM_08 is a novel singleton siphovirus with high host specificity for *Erwinia pyrifoliae***

Sang Guen Kim[1], Eunjung Roh[2], Jungkum Park[2], Sib Sankar Giri[1], Jun Kwon[1], Sang Wha Kim[1], Jung Woo Kang[1], Sung Bin Lee[1], Won Joon Jung[1], Young Min Lee[1], Kevin Cho[1], Se Chang Park[1*]

[1] Laboratory of Aquatic Biomedicine, College of Veterinary Medicine and Research Institute for Veterinary Science, Seoul National University, Seoul 08826, Republic of Korea

[2] Crop Protection Division, National Institute of Agriculture Sciences, Rural Development Administration, Wanju 55365, Republic of Korea

*Corresponding author: Email: parksec@snu.ac.kr; Phone: 82 2 880 1282; Fax: 82 2 880 1213

**Table S1** Functional categories of the identified open reading frames (ORFs) in pEp_SNUABM_08.

| Functional group | Locus tag | Identified protein | Protein ID | Related bacteriophage | Coverage (%) | Identity (%) |
|---|---|---|---|---|---|---|
| Nucleotide regulation | pEpSNUABM08_01 | Putative primase | QEQ94748.1 | *Salmonella* phage FSL SP-019 | 97 | 37.1 |
| Hypothetical protein | pEpSNUABM08_02 | Hypothetical protein | QEQ94749.1 | N/A[a] | N/A | N/A |
| Hypothetical protein | pEpSNUABM08_03 | Hypothetical protein | QEQ94750.1 | N/A | N/A | N/A |
| Hypothetical protein | pEpSNUABM08_04 | Hypothetical protein | QEQ94751.1 | N/A | N/A | N/A |
| Nucleotide regulation | pEpSNUABM08_05 | Cas4-like protein | QEQ94752.1 | *Burkholderia* phage AH2 | 95 | 39.37 |
| Hypothetical protein | pEpSNUABM08_06 | DUF2815 domain-containing protein | QEQ94753.1 | *Providencia* phage Redjac | 85 | 37.24 |
| Nucleotide regulation | pEpSNUABM08_07 | Putative DNA polymerase I | QEQ94754.1 | *Salmonella* virus FSLSP088 | 95 | 48.02 |
| Nucleotide regulation | pEpSNUABM08_08 | VRR-NUC domain-containing protein | QEQ94755.1 | *Burkholderia* phage AH2 | 84 | 39.33 |
| Nucleotide regulation | pEpSNUABM08_09 | Putative DEAD-like helicase | QEQ94756.1 | *Achromobacter* phage phiAxp-2 | 91 | 41.88 |
| Packaging | pEpSNUABM08_10 | Putative terminase small subunit | QEQ94757.1 | *Proteus* phage PM87 | 90 | 38.07 |
| Packaging | pEpSNUABM08_11 | Putative terminase large subunit | QEQ94758.1 | *Proteus* phage PM87 | 98 | 54.91 |
| Structure | pEpSNUABM08_12 | Putative head completion protein | QEQ94759.1 | *Klebsiella* phage Seifer | 87 | 49.38 |
| Structure | pEpSNUABM08_13 | Putative portal protein | QEQ94760.1 | *Serratia* phage JS26 | 95 | 52.01 |
| Structure | pEpSNUABM08_14 | Putative capsid assembly protease | QEQ94761.1 | *Serratia* phage JS26 | 94 | 43.29 |
| Structure | pEpSNUABM08_15 | Putative head decoration | QEQ94762.1 | *Burkholderia* phage AH2 | 94 | 42.17 |

| | | | | | | |
|---|---|---|---|---|---|---|
| | | protein | | | | |
| Structure | pEpSNUABM08_16 | Putative major capsid protein | QEQ94763.1 | *Klebsiella* phage Seifer | 96 | 42.41 |
| Hypothetical protein | pEpSNUABM08_17 | Hypothetical protein | QEQ94764.1 | *Serratia* phage JS26 | 98 | 33.59 |
| Structure | pEpSNUABM08_18 | Putative head-tail joining protein | QEQ94765.1 | *Salmonella* phage KFS-SE1 | 99 | 50.28 |
| Structure | pEpSNUABM08_19 | Putative tail completion protein | QEQ94766.1 | *Proteus* phage PM87 | 96 | 54 |
| Structure | pEpSNUABM08_20 | Putative tail tube terminator protein | QEQ94767.1 | *Enterobacter* phage Enc34 | 95 | 38.18 |
| Structure | pEpSNUABM08_21 | Ig-like domain-containing tail protein | QEQ94768.1 | *Salmonella* virus SPN19 | 82 | 42.9 |
| Structure | pEpSNUABM08_22 | Putative tail assembly chaperone | QEQ94769.1 | *Klebsiella* phage Seifer | 72 | 31.03 |
| Hypothetical protein | pEpSNUABM08_23 | Hypothetical protein | QEQ94770.1 | Serratia phage JS26 | 81 | 46.15 |
| Structure | pEpSNUABM08_24 | Putative tape measure protein | QEQ94771.1 | *Salmonella* virus iEPS5 | 82 | 32.71 |
| Structure | pEpSNUABM08_25 | Putative lambda tail tip M like protein | QEQ94772.1 | Unidentified phage | 95 | 35.82 |
| Structure | pEpSNUABM08_26 | Putative lambda tail tip L like protein | QEQ94773.1 | Unidentified phage | 99 | 46.91 |
| Structure | pEpSNUABM08_27 | Putative lambda tail tip I like protein | QEQ94774.1 | *Burkholderia* phage BcepNazgul | 76 | 56.45 |
| Hypothetical protein | pEpSNUABM08_28 | Hypothetical protein | QEQ94775.1 | *Xanthomonas* phage Xoo-sp2 | 83 | 36.67 |
| Structure | pEpSNUABM08_29 | Putative lambda tail tip J like protein | QEQ94776.1 | *Salmonella* phage Season12 | 92 | 40.46 |

| | | | | | | |
|---|---|---|---|---|---|---|
| Hypothetical protein | pEpSNUABM08_30 | Hypothetical protein | QEQ94777.1 | *Salmonella* virus FSLSP088 | 99 | 38.62 |
| Hypothetical protein | pEpSNUABM08_31 | Hypothetical protein | QEQ94778.1 | *Enterobacter* phage Enc34 | 97 | 32.01 |
| Hypothetical protein | pEpSNUABM08_32 | Hypothetical protein | QEQ94779.1 | *Proteus* phage PM87 | 99 | 38.79 |
| Hypothetical protein | pEpSNUABM08_33 | Hypothetical protein | QEQ94780.1 | *Salmonella* virus Chi | 99 | 49.86 |
| Hypothetical protein | pEpSNUABM08_34 | Hypothetical protein | QEQ94781.1 | *Salmonella* phage 118970_sal1 | 44 | 31.64 |
| Hypothetical protein | pEpSNUABM08_35 | Hypothetical protein | QEQ94782.1 | Serratia phage JS26 | 100 | 28.44 |
| Hypothetical protein | pEpSNUABM08_36 | Hypothetical protein | QEQ94783.1 | *Salmonella* phage Season12 | 82 | 23.99 |
| Hypothetical protein | pEpSNUABM08_37 | Hypothetical protein | QEQ94784.1 | N/A | N/A | N/A |
| Lysis | pEpSNUABM08_38 | Putative virion protein (lysis protein A like) | QEQ94785.1 | *Salmonella* phage FSL SP-099 | 97 | 63.52 |
| Lysis | pEpSNUABM08_39 | Hypothetical protein (Rz-like) | QEQ94786.1 | *Providencia* phage Redjac | 96 | 35.44 |
| Hypothetical protein | pEpSNUABM08_40 | Hypothetical protein | QEQ94787.1 | *Enterobacter* phage Enc34 | 69 | 50.85 |
| Hypothetical protein | pEpSNUABM08_41 | Hypothetical protein | QEQ94788.1 | N/A | N/A | N/A |
| Hypothetical protein | pEpSNUABM08_42 | Hypothetical protein | QEQ94789.1 | *Escherichia* phage vB_EcoM_ECO1230-10 | 25 | 56.98 |
| Hypothetical protein | pEpSNUABM08_43 | Hypothetical protein | QEQ94790.1 | N/A | N/A | N/A |
| Nucleotide regulation | pEpSNUABM08_44 | Putative N-6-adenine-methyltransferase | QEQ94791.1 | *Klebsiella* phage Seifer | 98 | 45.64 |
| Hypothetical protein | pEpSNUABM08_45 | Hypothetical protein | QEQ94792.1 | N/A | N/A | N/A |
| Hypothetical protein | pEpSNUABM08_46 | Hypothetical protein | QEQ94793.1 | N/A | N/A | N/A |
| Hypothetical protein | pEpSNUABM08_47 | Hypothetical protein | QEQ94794.1 | N/A | N/A | N/A |
| Hypothetical protein | pEpSNUABM08_48 | Hypothetical protein | QEQ94795.1 | *Proteus* phage PM87 | 79 | 38.75 |
| Hypothetical protein | pEpSNUABM08_49 | Hypothetical protein | QEQ94796.1 | Serratia phage JS26 | 46 | 32.32 |
| Hypothetical protein | pEpSNUABM08_50 | Hypothetical protein | QEQ94797.1 | Serratia phage JS26 | 35 | 39.62 |
| Structure | pEpSNUABM08_51 | Putative tail fiber protein | QEQ94798.1 | *Klebsiella* phage KPN N137 | 76 | 43.33 |

| | | | | | | |
|---|---|---|---|---|---|---|
| Nucleotide regulation | pEpSNUABM08_52 | Putative exonuclease | QEQ94799.1 | *Enterobacter* phage Enc34 | 66 | 28.67 |
| Hypothetical protein | pEpSNUABM08_53 | Hypothetical protein | QEQ94800.1 | N/A | N/A | N/A |
| Hypothetical protein | pEpSNUABM08_54 | Hypothetical protein | QEQ94801.1 | N/A | N/A | N/A |
| Hypothetical protein | pEpSNUABM08_55 | Hypothetical protein | QEQ94802.1 | *Serratia* phage JS26 | 95 | 35.51 |
| Hypothetical protein | pEpSNUABM08_56 | Hypothetical protein | QEQ94803.1 | N/A | N/A | N/A |
| Hypothetical protein | pEpSNUABM08_57 | Hypothetical protein | QEQ94804.1 | N/A | N/A | N/A |
| Hypothetical protein | pEpSNUABM08_58 | Hypothetical protein | QEQ94805.1 | N/A | N/A | N/A |
| Nucleotide regulation | pEpSNUABM08_59 | Putative cytosine C5-specific methylase | QEQ94806.1 | *Escherichia* phage K1-ind(2) | 97 | 46.4 |
| Hypothetical protein | pEpSNUABM08_60 | Hypothetical protein | QEQ94807.1 | N/A | N/A | N/A |
| Hypothetical protein | pEpSNUABM08_61 | Hypothetical protein | QEQ94808.1 | *Klebsiella* phage YMC16/01/N133_KPN_BP | 80 | 37.97 |
| Hypothetical protein | pEpSNUABM08_62 | Hypothetical protein | QEQ94809.1 | N/A | N/A | N/A |
| Hypothetical protein | pEpSNUABM08_63 | Hypothetical protein | QEQ94810.1 | *Klebsiella* phage KPN N137 | 57 | 53.41 |
| Hypothetical protein | pEpSNUABM08_64 | DUF1382 domain-containing protein | QEQ94811.1 | N/A | N/A | N/A |
| Hypothetical protein | pEpSNUABM08_65 | Hypothetical protein | QEQ94812.1 | *Klebsiella* phage YMC16/01/N133_KPN_BP | 56 | 38.24 |
| Hypothetical protein | pEpSNUABM08_66 | Hypothetical protein | QEQ94813.1 | *Pseudomonas* phage Psp6 | 22 | 50 |
| Nucleotide regulation | pEpSNUABM08_67 | Putative DnaQ-like exonuclease | QEQ94814.1 | *Pectobacterium* phage MA11 | 76 | 39.06 |
| Hypothetical protein | pEpSNUABM08_68 | Hypothetical protein | QEQ94815.1 | N/A | N/A | N/A |
| Hypothetical protein | pEpSNUABM08_69 | Hypothetical protein | QEQ94816.1 | *Salmonella* phage BSPM4 | 72 | 68.75 |
| Hypothetical protein | pEpSNUABM08_70 | Hypothetical protein | QEQ94817.1 | *Salmonella* phage FSL SP-124 | 98 | 52.2 |
| Hypothetical protein | pEpSNUABM08_71 | Hypothetical protein | QEQ94818.1 | *Salmonella* phage 118970_sal1 | 99 | 53.03 |

| | | | | | | |
|---|---|---|---|---|---|---|
| Hypothetical protein | pEpSNUABM08_72 | Hypothetical protein | QEQ94819.1 | N/A | N/A | N/A |
| Hypothetical protein | pEpSNUABM08_73 | Hypothetical protein | QEQ94820.1 | N/A | N/A | N/A |
| Hypothetical protein | pEpSNUABM08_74 | Hypothetical protein | QEQ94821.1 | N/A | N/A | N/A |
| Hypothetical protein | pEpSNUABM08_75 | Hypothetical protein | QEQ94822.1 | *Escherichia* phage vB_EcoM-Ro121c4YLVW | 43 | 46.22 |
| Hypothetical protein | pEpSNUABM08_76 | Hypothetical protein | QEQ94823.1 | N/A | N/A | N/A |
| Hypothetical protein | pEpSNUABM08_77 | Hypothetical protein | QEQ94824.1 | N/A | N/A | N/A |
| Hypothetical protein | pEpSNUABM08_78 | Hypothetical protein | QEQ94825.1 | N/A | N/A | N/A |
| Hypothetical protein | pEpSNUABM08_79 | Hypothetical protein | QEQ94826.1 | N/A | N/A | N/A |

[a]N/A, Not available.

**Fig. S1** ERIC-PCR genotyping of Erwinia spp. used in this study for host range analysis; M, 1kb plus size marker;-, negative control; 1-26, *E. amylovora* strains; 27-51, *E. pyrifoliae* strains; 1, YKB 14715; 2, YKB 14740; 3, YKB 14742; 4, YKB 14748; 5, YKB 14750; 6, RA0030; 7, RA0031; 8, RA0032; 9, RA0033; 10, RA0034; 11, RA0035; 12, RA0041; 13, RA0042; 14, RA0043; 15, RA0044; 16, RA0045; 17, RA0051; 18, RA0052; 19, RA0053; 20, RA0054; 21, RA0055; 22, RA0062; 23, RA0063; 24, RA0064; 25, RA0065; 26, RA0066; 27, KACC13945; 28, KACC13946; 29, KACC13948; 30, KACC13949; 31, KACC13952; 32, RP0100; 33, RP0101; 34, RP0102; 35, RP0103; 36, RP0104; 37, RP0105; 38, RP0108; 39, RP0109; 40, RP0110, 41, RP0111; 42, RP0112; 43, RP0113; 44, RP0114; 45, RP0115; 46, RP0116; 47, RP0117; 48, RP0118; 49, RP0119; 50, RP0120; 51, RP0121.

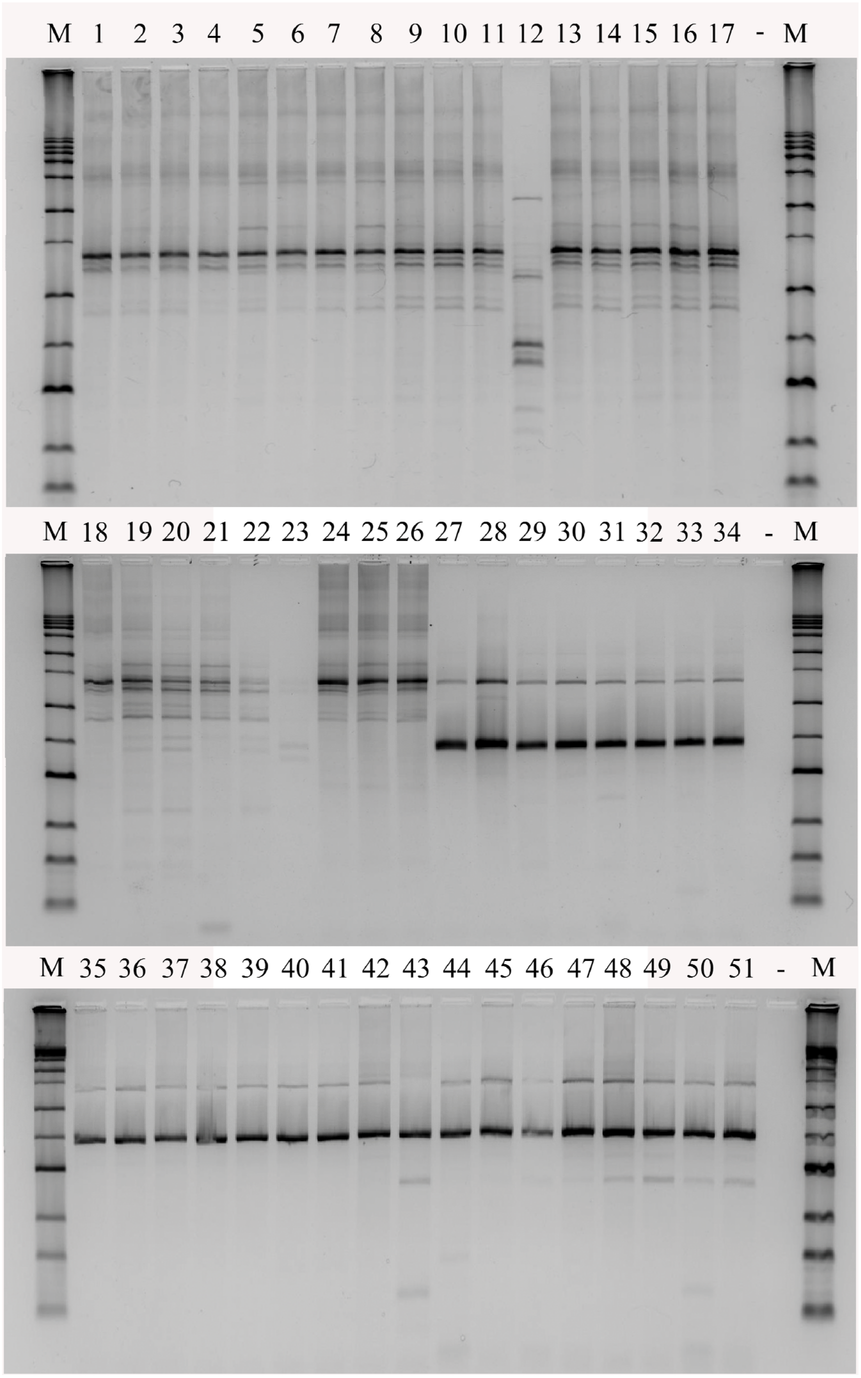

**Fig. S2** Comparative genome analysis of *Erwinia* phage pEp_SNUABM_08 with *Salmonella* phage Chi. The tBLASTx comparison result was represented in blue and red by their direction and the intensity of color represents the % identity.

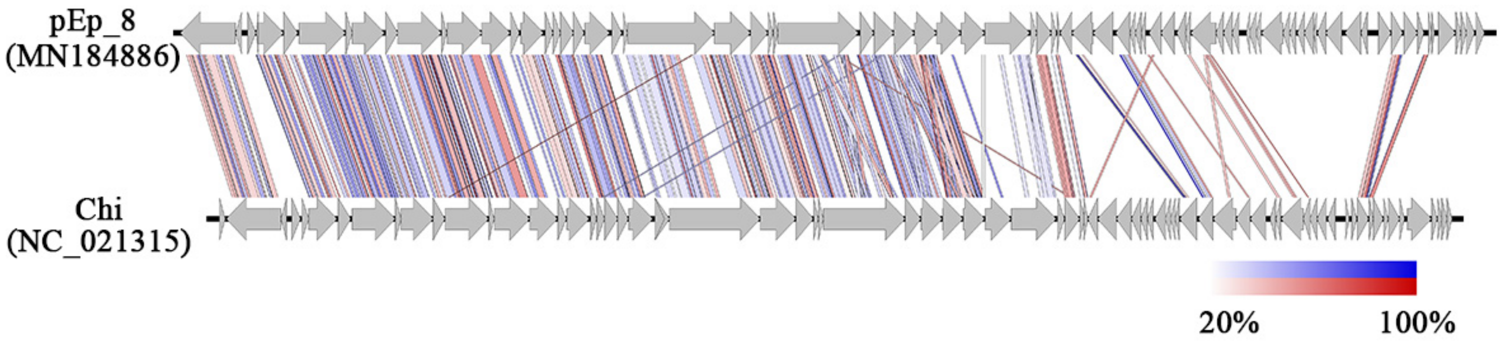


.
